# Benchmark Survey on Content Development and Key Performance Indicators: A phactMI Benchmarking Survey Update

**DOI:** 10.1007/s43441-026-00999-9

**Published:** 2026-06-15

**Authors:** Robert Tamburri, Mary K. Sendi, Anja Glaetzer, Jennifer Garas, Mary Mehrabian, Susan Wnorowski, Evelyn R. Hermes-DeSantis

**Affiliations:** 1 phactMI, 5931 NW 1st Place, Gainesville, FL 32607 USA; 2 Johnson and Johnson, Robbinsville, PA USA; 3 formerly Pfizer Inc.,, New York, NY USA; 4 Sanofi, Bridgewater, NJ USA; 5 BristolMyers Squibb, Plainsboro, NJ USA; 6 Eversana, Chicago, IL USA; 7 Bridgewater, NJ USA

**Keywords:** Medical Information, Benchmarking, Content Development, Metrics, KPIs

## Abstract

**Objective:**

Medical Information plays a critical role in delivering accurate, evidence-based responses to unsolicited requests. At the heart of the response is the content developed by Medical Information. Additionally, the metrics and key performance indicators (KPIs) surrounding content development and delivery are necessary to gauge the utilization and impact of the content. Because the landscape has evolved significantly since the last survey in 2018, the need to re-examine content development approaches and KPIs is warranted. The objective of this benchmarking survey was to assess the evolving approach and tactics of content development and utilization of KPIs of phactMI member companies with a comparison to 2018 where applicable.

**Methods:**

In May 2024, an electronic survey containing 71 closed and open-ended questions was distributed to 36 phactMI member companies. The survey included demographics, development of specific content, scientific response documents (SRDs), custom response documents, and metrics and KPI. Results were analyzed with descriptive statistics.

**Results:**

Medical Information teams primarily develop content for HCPs (90%) versus patients (10%). Disease state content creation was more common among small/midsize companies (36%) than large companies (18%), though requests for such content were infrequent. Similar to 2018, SRDs remain predominantly traditional written documents (66%), with emerging use of frequently asked questions, infographics, HTML, and slide decks. Most companies (85%) maintain centralized SRD development, but the proportion without target timelines for SRD development increased from 37% in 2018 to 72% in 2024. AI adoption is in early stages, with 3 companies actively using it and 18 exploring applications, primarily for writing and paraphrasing. Omnichannel integration is reported by 59% of companies, and custom response documents account for 25% of inquiries. Similar to 2018, the most common KPIs in medical information departments include inquiry volume, turnaround time, and document updates. There is a growing emphasis on customer satisfaction and digital engagement. HCPs most frequently use email (52%) and phone (29%) for information, while digital channels remain underutilized.

**Conclusion:**

This benchmarking survey highlights the need for ongoing innovation, strategic alignment, and robust quality assurance in Medical Information content development and KPI reporting. While many things are similar to 2018, customer expectations continue to evolve and technology has advanced. Organizations must balance operational efficiency with personalized engagement, while ensuring compliance and ethical standards. For the future, the three areas to prioritize are establishing non-traditional content development formats, processes, and workflows; expanding digital and mobile optimization; and strengthening governance around AI and content reuse.

**Supplementary Information:**

The online version contains supplementary material available at https://doi.org/10.1007/s43441-026-00999-9.

## Introduction

Medical Information departments within the pharmaceutical industry play a critical role in providing healthcare professionals (HCPs) and patients with accurate, timely, and scientifically balanced information. As the healthcare landscape continues to evolve—driven by heightened expectations for transparency, expanded digital engagement, and growing reliance on real-world evidence—the strategic importance of Medical Information continues to grow [1–4]. Additionally, technology is advancing rapidly and the use of AI to assist with developing scientific responses is now being piloted and implemented [4]. To meet these changes and demands, Medical Information teams must continuously evaluate and optimize their operational practices, including content development methods, workflows, and performance metrics.

The Pharma Collaboration for Transparent Medical Information (phactMI) is a consortium of Medical Information leaders from across the industry, dedicated to advancing the practice of Medical Information through benchmarking, innovation, and shared best practices. A 2018 phactMI benchmarking survey examined content creation and key performance indicators (KPIs) within Medical Information Departments from 27 US pharmaceutical/biopharmaceutical companies [2, 3]. Byun et al. highlight ongoing opportunities to showcase and elevate the value of Medical Information, emphasizing the importance of meeting the evolving expectations of customers. Additionally, they advocated for continuous innovation in how medical information is delivered, reinforcing its role in supporting patient care [2]. 

The industry landscape has undergone significant changes since the original 2018 benchmark survey. The objective of this benchmarking survey was to assess the evolving approach and tactics of content development and the associated use of KPIs of phactMI member companies and to compare the results, where applicable, to the 2018 benchmark survey.

## Materials and Methods

A working group of 13 phactMI volunteers built and deployed a digital survey via the Alchemer.com [Louisville, CO] survey platform. The survey was piloted by members of the working group to assess clarity and programming. The survey was distributed to a single contact at each of the 36 US bio/pharmaceutical member companies of phactMI in May 2024. Participants had 90 days to complete the survey, with reminders sent periodically. The system autosaved responses, enabling participants to resume the survey at any time.

The cross-sectional survey included 71 closed and open-ended questions (Appendix 1) which expanded upon the original 2018 survey of 38 questions on these topics. Categories included demographics, development of specific content (disease state/patient), development of scientific response documents (SRDs), development of custom response documents, and metrics and KPIs.

The survey consisted of five sections focusing on:

1. *Company background information* based on the previous (2023) calendar year, including the size and coverage of the Medical Information department.
2. *Development of specific content*. Questions assessed specific types of information such as disease state and patient information provided to customers.
3. *Scientific response documents*. Questions assess various aspects of the development of SRDs.
4. *Custom response documents*. Questions assessed various aspects of the development of custom response documents.
5. *Metrics and KPI*. Questions assessed specific metrics and KPIs measured.

The survey used skip logic to omit irrelevant questions depending on earlier answers. For example, if no disease-state responses were disseminated, additional questions on disease-state responses were skipped. Information was de-identified and analyzed in aggregate.

Descriptive statistics, Chi square, and Mann-Whitney U tests were used to assess the data as appropriate. Missing data was accounted for in sample size reporting for each question as all questions were optional.

## Results

### Organizational Demographics

A total of 35 of 36 (97%) phactMI member companies took part in the survey with each company responding to specific questions based on their involvement. Table 1 summarizes the demographics stratified by three company-size categories based on 2023 revenue: small companies earning less than $10 billion, mid-size companies earning $10–28 billion, and large companies earning more than $28 billion. The number of companies that answered the specific question based on their scope of work is represented by the denominator.

The average number of internal Medical Information employees was 31 full time equivalents (FTEs) (range 2–200+) while external employees accounted for an average of 21 FTEs (range 0–130). On average, a Medical Information department supports 64 products (range 4–524). When evaluated in terms of life cycle of a drug, a Medical Information department supports an average of 9 products pre-launch (pre-launch to six months post launch; range 0–160); 24 current or active products (range 1–320); 24 mature or established products (range 0–275); and 11 pipeline products (range 0–100).

A majority (62%, 21/34) of Medical Information teams that responded to this question were organized by therapeutic area. Centralization of content development was seen in a majority (82%, 28/34) of companies, with the rest indicating a decentralized model. Additionally, no company with decentralized content development considered themselves global only.

**Table 1:** Demographics

	Overall *N*	Large company *N* (%)	Midsize company *N* (%)	Small company *N* (%)
Total responses	35	12 (34)	9 (26)	14 (40)
Internal FTEs (excluding call center)	Avg: 31	Avg: 45	Avg: 48	Avg: 7
	Range: 2-200	Range 5-100	Range: 4-200	Range: 2–15
External FTEs (excluding call center)	Avg: 21	Avg: 42	Avg: 15	Avg: 5
	Range 0–130	Range: 0–130	Range: 0–100	Range: 0–35
Department coverage				
US:	4 (11)	1 (8)	1 (11)	2 (14)
US/Global:	25 (71)	10 (83)	7 (78)	8 (57)
Global:	6 (17)	1 (8)	1 (11)	4 (29)
Department organization (*n* = 34)				
Therapeutic area	21(62)	8 (70)	4 (44)	9 (64)
Franchise	1 (3)	0 (0)	0 (0)	1 (7)
Product	4 (12)	1 (10)	2 (22)	1 (7)
Other*	8 (24)	2 (18)	3 (33)	3 (21)

Small companies (<$10 billion), mid-size companies ($10–28 billion), large companies (> $28 billion) based on 2023 revenue

Other: Regionally and functionally; medical business unit, both therapeutic area and product; products for major products and therapeutic area for lower volumes; depends on country and number of resources – majority are by therapeutic area, but in some areas colleagues work on everything; global is by therapeutic area, US is cross therapeutic area; region; organized based on tumor type

Appendix 2 provides a summary of survey findings across large, midsize, and small pharmaceutical companies while Appendix 3 presents a comparison of the 2024 results with corresponding data from 2018.

## Content Development

### Development of Specific Content

Based on the responses from 33 companies, Medical Information Teams reported that on average 90% of their content was developed for HCPs (range 40–100%) compared with 10% for patients (range 0–60%). Interestingly, Medical Information with a US-only focus reported creating a higher proportion of patient-focused content compared with Global-only teams (mean 22% [range 2–60%, *n* = 4] vs. 8% [range 0–20%, *n* = 6]).

Only 10 companies reported creating disease state content, highlighting that this activity is limited to a minority of survey respondents. When analyzed by organizational scope, Global only pharmaceutical companies create more disease state information (50%, 3/6) compared to US only companies (25%, 1/4).

Of the 10 companies producing disease state content, 70% (7/10) report developing these materials exclusively for HCPs. Three companies indicated that these responses were for patients: 1 only creates these responses for patients and 2 companies reported they create disease state responses for both audiences.

**Fig. 1:**
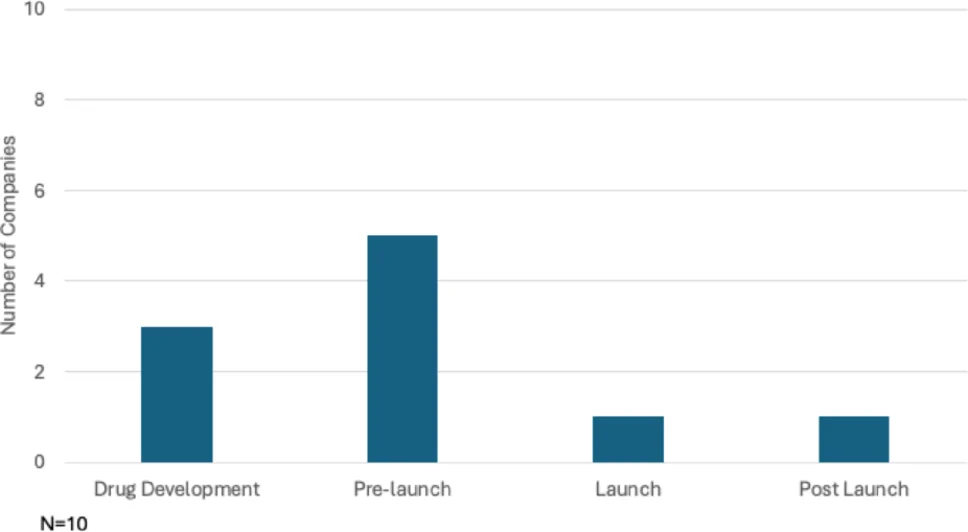
Timing of creation of disease state only responses *n* = 10. Drug development - When the drug is in development (prior to the pre-launch phase). Pre-launch - just prior to expected FDA approval. Launch - from approval to 6 months post approval. Post-launch - any time after 6 months post approval

Among the 10 pharmaceutical companies that prepared disease state response documents, the mean number developed was 9 (range 3–35). Disease state responses were infrequently used, with 70% (7/10) of the companies reporting usage of 1–5 time per month. Of 10 companies with disease state documents, 50% (5/10) create these during the pre-launch phase of the drug. (Fig. 1) All 10 companies created traditional (static) disease state SRDs, and 80% (8/10) of these companies also used one or more alternative formats including interactive SRDs (40%, 4/10), infographics (40%, 4/10), video (10%, 1/10) or a citation list (10%, 1/10). The most common reasons for creating disease state response documents were to answer specific unsolicited questions and to provide disease state background. The majority of companies (78%, 7/9) use Medical Information for the development of disease state information.

## Scientific Response Documents

91% (30/33) of companies reported that internal Medical Information staff are responsible for SRD development, with full ownership seen more commonly in small and midsize companies. This was down slightly from 100% (27/27) in 2018. Larger companies were more likely to involve vendors and other departments (e.g., Scientific Communications, Contact Centers). Since 2018 the reliance on external vendors to create and revise SRDs increased from 19% (5/27) to 55% (18/33).

**Fig. 2:**
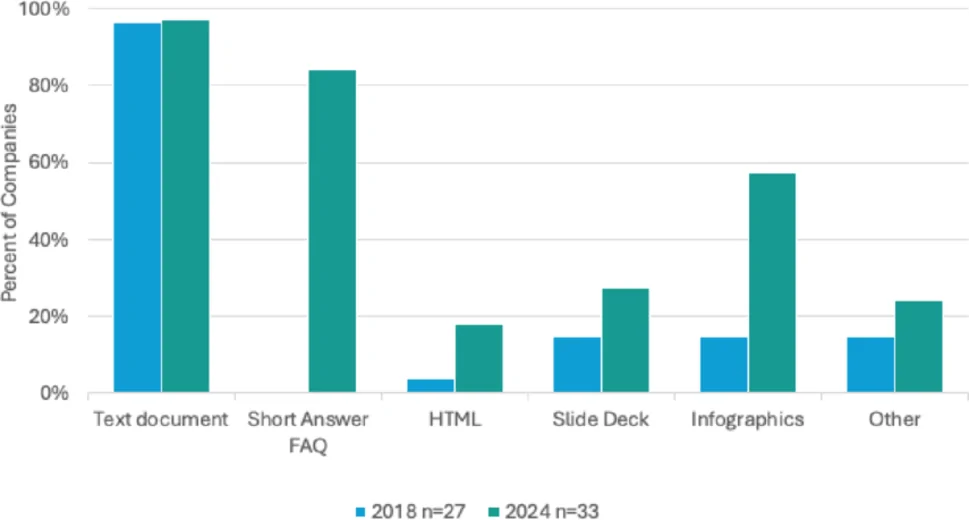
Format of Scientific Response Documents utilized in 2024 (*n* = 33) vs. 2018 (*n* = 27). Note: Text document includes both all text and text with graphics. In 2024, 2 companies did not respond to this question. Note: Text document includes both all text and text with graphics. In 2024, 2 companies did not respond to this question

Among the 33 responding companies, SRD were commonly formatted as traditional written documents with charts and text (66%). Other formats included traditional text-only documents (17%), FAQ formats (10%), HTML (3%), slide decks (3%), and infographics (2%). An HTML-based SRD offers advantages such as semantically structure, screen-reader compatibility, accessible metadata, and improved handling of figures. On average, SRDs were 5 pages in length (range 2–15 pages, mode 3 pages), although document length varied by company size with large averaging 4.1 pages, midsize 6.5 pages, and small 5.5 pages. Compared with 2018, companies increasingly developed short-answer FAQ formats and adopted more innovative presentation formats such as infographics, slide decks, and HTML-based SRDs (Fig. 2)

While most SRDs remained static, a trend toward adapting content for mobile platforms (24%, 8/33) was observed in 2024, particularly among larger companies compared to small and midsize companies (45%, 5/11 vs. 14% 3/22, *p* < 0.05, X^2^ 4.0425). However, most of the companies have not adapted SRDs for mobile content.

Of the 33 companies responding, 85% (28/33) report continuing to use a centralized SRD development process, consistent with findings from 2018. Centralized models varied and included Global Medical Information-developed documents (Global Response Documents or GRDs) that were localized by countries; US Medical Information-developed documents localized globally; and other hybrid approaches. An average of 60% of parent SRDs are localized or repurposed by US Medical Information (*n* = 22, range 0–100%, mode 100%), noting that in some cases the parent document is from US Medical Information. Localization increased with company size, with 88% (7/8) of large companies localizing over 60% of documents compared to 50% (7/14) of midsized and small companies. Content most frequently requiring localization included labeling information, disclaimers and guidelines, summaries, and document format. Large and small companies localized similar content elements, whereas midsize companies generally did not modify document format during the localization process.

**Fig. 3:**
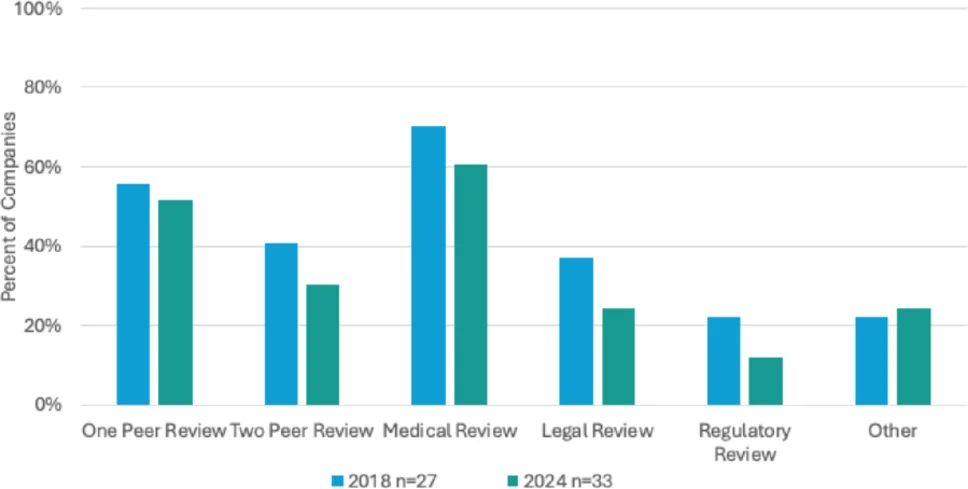
Type of review of SRDs conducted in 2024 (*n* = 33) vs. 2018 (*n* = 27). Note: In 2024, 8 companies did not respond to this question

Few companies reported setting target timelines for SRD creation, with the proportion of companies without a defined target timeline doubling from 37% (10/27) in 2018 to 72% (23/32) in 2024. A similar trend was observed for SRD revisions and updates, where the percentage of companies not setting a target timeline increased from 37% (10/27) in 2018 to 59% (19/32) in 2024. SRDs were typically reviewed by Medical Information peers (82%, 27/33), Medical colleagues (94%, 31/33), with fewer reviews involving legal (24%, 8/33), and regulatory (12%, 4/33). Most companies described a streamlined peer-review process, with 63% (17/27) conducting a single peer review focused on data accuracy and high-level context, while 37% (10/27) reported two separate peer reviews addressing these components. Medical reviews were primarily conducted by Medical Directors (50%,13/26), followed by Medical Information (23%, 6/26), other medical colleagues (19%, 5/26), and Therapeutic Heads (8%, 2/26). Overall, fewer review resources were involved compared with 2018 (Fig. 3) Review timelines varied widely depending on SRD type, length, complexity, and review requirements. A range of 0.5 h-20 days was reported, with the average of 21 h.

Most companies applied an off-label designation to SRDs as appropriate (66%, 21/32), consistent with findings from 2018. The review and approval process did not differ between on-label vs. off-label SRDs.

Expiration timelines for SRDs varied by topic, with 1- to 2-year review periods being most common, regardless of company size or development model. Most companies reviewed new product SRDs on a yearly basis (87%, 27/31). These findings were consistent with 2018, when 93% (24/ 26) of companies reported a 1- to 2-year expiration date for SRDs overall.

### Scientific Response Document Innovation

SRDs typically included summary and reference sections (each 94%, 31/33); however, company contact information was rarely included (9%, 3/33), which may limit standalone usability when documents are reviewed without an accompanying cover letter. Most companies included hyperlinks to external sources (73%, 24/33) and internal document sections  (64%, 21/33), as well as disclaimers (61%, 20/33), advanced graphics (55%, 18/33), and tables of contents (42%, 14/33). These enhanced and innovative features - particularly hyperlinks, embedded videos, and table of contents - were more commonly implemented by large companies than by small or midsized companies. Compared to 2018, the use of hyperlinks (73% vs. 67%), advanced graphics (55% vs. 30%) and the table of contents (42% vs. 15%) increased notably in 2024.

Use of artificial intelligence (AI) in SRD development remains limited, with 9% (3/33) of companies reporting current use and 55% (18/33) reporting investigating or testing. Among the 64% (21/33) of companies using or exploring AI for content development, human validation was consistently required. Of these companies, 83% (15/18) focused AI use on content drafting and 72% (13/18) on paraphrasing, while large companies reported broader applications including image and visualization creation, analytic support and business insight generation. Additional AI use cases included targeted testing of paraphrasing, translations, and table generation.

Medical Information content was increasingly integrated into omnichannel strategies, reported by 59% (19/32) of companies, particularly among large companies (82%, 9/11), those with a U.S. presence (62%, 16/26), and decentralized organizations (100%, 5/5). Content was most commonly delivered via Medical Information websites (91%, 29/32) and Medical Information Contact Centers (MICC) (81%, 26/32), with additional use of field materials, tools such as calculators, chatbots, live chat, interactive voice response, and social media. Modular content strategies were reported by 19% (6/32) of companies, all of which were large and centralized.

Just over half of companies (56%, 18/32) reported incorporate accessibility features into their content, including color considerations (56%, 10/18), alternative text for images (44%, 8/18), font selection (39%, 7 /18), reader compatibility (6%, 1/18), and spacing (6%, 1/18). Although accessibility was not addressed in the 2018 survey, these findings indicate a growing emphasis on inclusive content development.

Specialty SRDs were developed by 40% (4/35) of companies to address specific needs, including patient-focused response documents and digital enhanced content such as video and hyperlinks. Common topics included mechanism of action, dosing and administration, clinical trials, and safety, with digital formats used more frequently that patient-specific versions across all categories.

## Custom Response Documents

Custom response documents were developed when no SRD existed for a specific inquiry, ensuring accurate, compliant, and evidence‑based responses tailored to the requestor. Medical Information teams served as the primary developers of custom responses, a slight increase from 2018 (94%, 29/31 vs. 85%, 22/26). Among responding companies, an average of 25% of inquiries required a custom response (range: 0%-75%), with smaller companies reporting the highest proportion (33%) compared with midsized (22%) and large companies (19%).

**Fig. 4:**
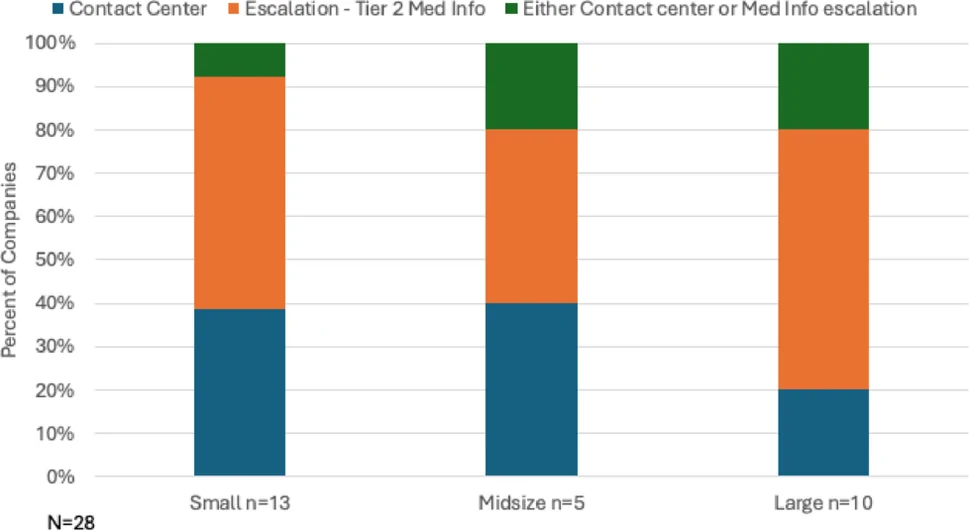
Dissemination of custom response documents. Note: 7 companies did not respond to this question (2 do not respond with custom response documents and skipped this question)

Of the companies describing response format, 66% (19/29) provided full letters supplemented with literature summaries, while 14% (4/29) offered a reference list only and 21% (6/29) used a mixed approach. Dissemination was predominantly managed by escalation or Tier 2 Medical Information support teams (54%, 15/28), followed by contact centers (32%, 9/28) or both (14%, 4/28) (Fig. 4)

Expiration practices for custom responses varied widely, ranging from one-time use to 18 months. The most common expiration period was 12 months (27%, 7/26), followed by one-time use (19%, 5/26), and 24 h (12%, 3/26). Smaller companies were more inclined to designate custom replies for one-time use only (45%, 5/11).

Target development timelines ranged from 2 to 5 h up to 3–5 days, with the most common target being within 48 h (24%, 6/25), 2–5 h (16%, 4/25) and within 72 h (16%, 4/25). Compared with 2018, development timelines increased significantly with most companies reporting targets greater than 20 h in 2024 (68%, 15/22 vs. 25%, 3/12; *Χ*^*2*^ 7.618. *p* < 0.05).

Custom responses were primarily created by Medical Information internal staff (97%, 29/30); with support from external contractors (33% 10/30) or contact center (23% 7/30). Similar to SRDs, most custom responses underwent peer review for data accuracy and contextual appropriateness (83%, 24/29), with additional medical review occurring in 41% (12/29) of cases; only 7% (2/29) reported no formal review.

## Metrics and KPIs

**Fig. 5:**
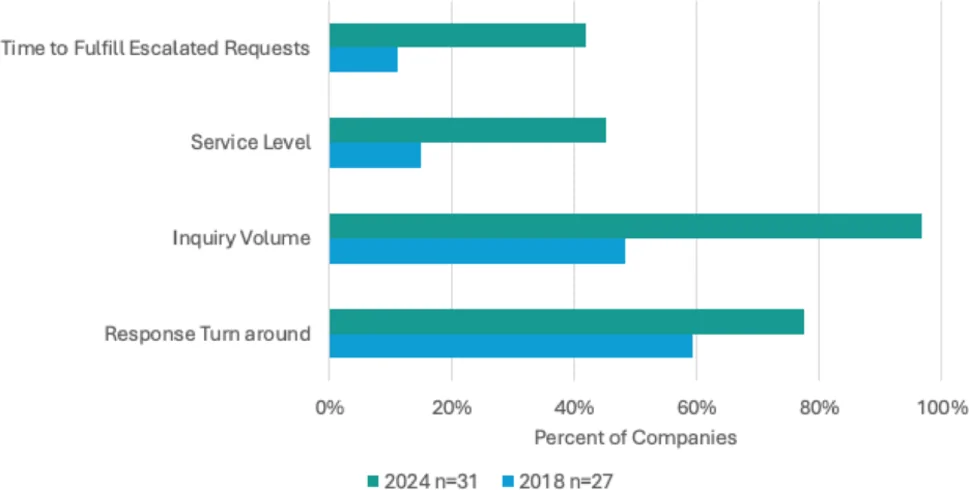
Top KPIs reported to senior leadership, comparison of 2024 (*n* = 31) to 2018 (*n* = 27). Note: In 2024, 4 companies did not respond to this question

Across surveyed pharmaceutical companies, 61% (19/31) differentiate between metrics and KPIs, with larger (64%, 7/11), midsized (71%, 5/7), and centralized (65%, 17/26) organizations more likely to make this distinction. Metrics were defined as broad measures of activity, while KPIs were described as strategic indicators monitored regularly and reported to leadership. The most commonly reported KPIs include inquiry volume (97%, 30/31), response turnaround time (77%, 24/31), number of documents created or updated (65%,20/31), topics or category of questions (55%, 17/31), escalation rate (55%, 17/31), contact types (55%, 15/31), time to fulfill escalated requests (55%, 15/31), and service level (45%, 14/31). These KPIs reflect operational efficiency, responsiveness, and content relevance. Centralized teams consistently report more KPIs across categories, including document localization and HCP satisfaction, compared to decentralized teams. A shift in the top KPIs reported to senior leadership was observed compared with 2018. (See Fig. 5)

Customer satisfaction query metrics were evaluated among the 21 companies that reported administering customer surveys. These metrics were primarily tracked through the Medical Information Contact Center, with 71% (15/21) of companies using this channel in 2024, up from 52% in 2018; surveys attached to written response documents declined (43%, 9/21 vs. 59%). However, the comparison is limited as only the percentage data was reported in 2018. The most common metrics and KPIs related to customer queries include inquiry volume (74%, 23/31), response turnaround (71%, 22/31), customer satisfaction surveys (68%, 21/31), service level (68%, 21/31), abandonment rate (58%, 18/31), and escalation rate (52%, 16/31).

**Fig. 6:**
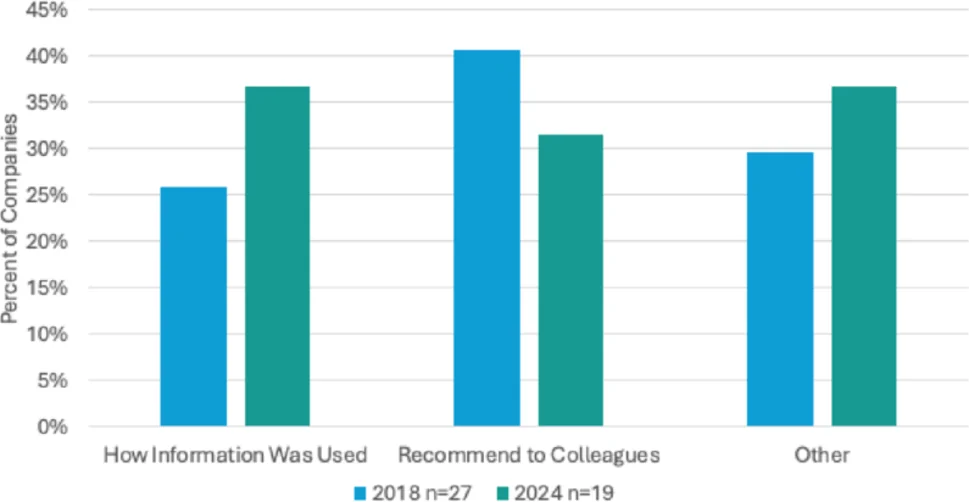
Customer satisfaction metrics measured, comparison of 2024 (*n* = 19) vs. 2018 (*n* = 27). Other 2018: Would call again, SLA met, response/materials relevant. Other 2024: Net Promoter score, sentiment analysis, trust assessment, impact to patient. Note: 2024: 21 companies collect customer satisfaction metrics, 2 did not respond to this question

Among companies accessing customer satisfaction metrics in greater detail, 53% (10/19) evaluated patient impact; 37% (7/19) assessed how information was used; 32% (6/19) measured likelihood to recommend to a colleague, and 37% (7/19) reported other measures. Additional metrics included sentiment analysis and trust assessment, each used by four companies (21%), while only two companies (11%) use Net Promoter score, representing a shift from 2018 (Fig. 6). Sentiment analysis was used to evaluate the tone and emotional perception of responses, helping organizations understand whether interactions were viewed positively, neutrally, or negatively. Trust assessment metrics were employed to gauge customers’ confidence in the accuracy, reliability, and credibility of the information provided.

While all companies surveyed HCPs (20/20), the proportion surveying both HCP and patients increased from 44% in 2018 to 60% in 2024. However, only 13% (4/32) measured patient impact through a survey-based question.

In an omnichannel environment, it is important to understand how various channels are used. The following are averages reported for both HCPs and patients. HCPs tended to rely on email (52%; range: 0–88%), verbal/phone (29%; 1–71%), digital medical information websites (9%; 0–69%), digital search engines (4%; 0–50%), chatbots (2%; 0–50%), voice bots (1%; 0–10%), and regular mail (1%; 0–6%) to receive information. Channel preferences differed for patients and caregivers, who primarily used verbal-phone (65%; 0–100%), followed by email (17%; 0–90%), digital medical information websites (2%; 0–45%), and digital search engines (4%; 0–75%).

Most companies (80%, 24/30) self-monitored performance to ensure metrics and KPIs were met. As quality assurance for Medical Information services were often conducted by multiple groups, monitoring was also performed by Quality Assurance teams (37%, 11/30), Pharmacovigilance (30%, 9/30), and Compliance (17%, 5/30). Within Medical Information Contact Center, 53% (17/32) conduct quality assurance on 5–10% of interactions, while 19% (6/32) monitored more than 10%, including four companies that reviewed up to 100% of interactions. Small and midsize companies more commonly reviewed 5–10% of interactions, whereas larger companies tended to use predefined sampling approached or smaller percentages.

When assessing the number of cases reviewed each month, most companies (84%, 27/32) did not cap the number of interactions, while 16% (5/32) applied caps ranging from 1 to 2 per week to 10–20 per month, depending on the therapeutic area. In most cases (94%, 29/31), quality assurance practices did not differ between general and escalated inquiries.

Digital metrics continued to gain importance, although adoption is uneven. Among 30 companies, 70% (21/30) track SRD access via Medical Information websites, 47% (14/30) track downloads, 33% (10/30) track digital engagement on website, and 33% (10/30) track SRD access on the phactMI site. However, only 31% (10/32) report using digital metrics to inform content development or channel strategy. These metrics supported decisions on SRDs retention, update, retirement, keyword optimization, and cross-functional planning.

While 31% (8/26) of companies can differentiate between internal and external users, only 16% (5/32) capture analytics on internal use of Medical Information assets, such as SRD usage by Field Medical or Medical Science Liaisons (MSLs). Overall, although foundational metrics such as inquiry volume and turnaround time were widely tracked, opportunities remain to expand digital and internal utilization metrics to support more strategic decision-making.

## Discussion

The 2024 benchmarking survey on content development and KPIs highlighted a dynamic and evolving landscape of Medical Information across pharmaceutical companies. Compared to 2018, findings demonstrated a clear shift towards more diverse content formats, increased use of digital tools, and a growing emphasis on operational efficiencies and strategic alignment.

While most content continued to be designed primarily for HCPs, patient‑focused content remains essential for promoting health literacy, shared decision‑making, and appropriate medication use, and continues to evolve as a key area of Medical Affairs activity [5]. Companies typically developed disease-state response documents during drug development or just before a product launch; however, these documents are infrequently requested. The need for disease state information was likely being met through alternative means such as medical education initiatives and non-pharmaceutical industry websites [6]. 

One notable trend was the increased reliance on external vendors for SRD creation, particularly among larger companies. The use of external vendors, especially for content creation, enable organizations to manage fluctuating inquiry volumes, maintain timely and accurate responses, and improved scalability, often leading to improved customer engagement. The decline in defined development timeline targets may reflect increasing complexity, expanded format diversity, greater integration of external vendors, or a strategic shift towards emphasizing more meaningful performance metrics. As content ranges from SRDs to FAQs to videos to infographics, maintaining consistent timeline for development and revision becomes more challenging. Some organizations may have intentionally presented flexibility to prioritize urgent updates while deferring lower priority revisions. Although this approach supported timely responses to high-priority needs, it introduces variability that could affect responsiveness and customer satisfaction, underscoring the need for continued monitoring.

Adoption of innovative formats, such as infographics, HTML-based content, and videos, signaled a shift towards more engaging and accessible information delivery. These formats enable more personalized, user-centered communications aligned with evolving preferences. However, format innovation alone was insufficient. To maximize impact, content needed to be optimized for use across multiple channels, including mobile-friendly platforms, to ensure accessibility, consistency, and relevance throughout the customer journey. Despite its potential to improve user experience, usability, and reach, mobile optimization remained underutilized. Similarly, although accessibility features were increasingly incorporated, implementation remained inconsistent across companies and should be expanded. Improving accessibility was important not only to align with recognized digital acceptability standards but also to support compliance with legal and regulatory expectations related to equitable information access for individuals with disabilities and to reflect emerging industry best practices. Ensuring the ongoing accuracy and reliability of reused content remains essential for risk mitigation. When custom responses were requested repeatedly conversion into

When custom response documents continued to play a critical role, particularly within smaller organizations, by addressing unique or infrequently requested inquiries. However, workflows for developing, reviewing, approving, and reusing these documents varied significantly. Reuse of custom content without sufficient oversight may introduce potential quality and regulatory risks. Decisions regarding re‑review were influenced by factors such as the timing of reuse, potential changes in evidence, and the level of oversight applied. In some cases, informal checks, such as updated literature assessments, were performed prior to reuse, though these steps were not fully captured in the survey. Ensuring the ongoing accuracy and reliability of reused content remained essential for risk mitigation. When custom responses were requested repeatedly, conversion into standard SRDs may have been appropriate; however, while this survey did not assess formal criteria for conversion, standardized processes were necessary to preserve content quality.

Metrics and KPIs continued to evolve, with increasing emphasis on customer satisfaction, digital engagement, and internal utilization. These insights supported the prioritization of strategic content development, identification of emerging trends, and confirmation of customer sentiment. For example, recurring inquiry patterns could indicate unmet data needs or opportunities for enhanced field education. Despite 59% of companies reporting omnichannel integration, only 31% report using digital metrics to inform content or channel strategy, indicating a gap between structural capability and data-driven execution. Limited differentiation between internal and external users further constrains actionable insights. This disconnect highlights opportunities for greater digital optimization to more fully realize the value of omnichannel strategies.

Integration of AI and omnichannel approaches remained in early stages. While promising, these technologies require robust governance frameworks, including human-in-the-loop validation to ensure accuracy, compliance, and ethical use. Effective AI governance depends on clear policies, ongoing risk‑management and audits, accountability structures, and training to promote responsible use. Recent publications and publicly available resources emphasized the importance of quality frameworks for AI implementation, including regulatory foundations, data traceability, protection, and verifiable outputs [4, 7–9]. 

Limitations.

Direct comparisons to the 2018 data may have been influenced by differences in how survey questions were worded and interpreted by respondents. In addition, the current survey expanded on the 2018 survey, resulting in some questions without a comparator data set. As no survey questions were mandatory, reporting includes varying denominators across data points. phactMI represents 36 US pharmaceutical companies, which provides valuable insights into current industry practices; however, the sample does not encompass the full range of organizations within the pharmaceutical sector and may not fully represent the broader industry landscape.

## Conclusion

This benchmarking survey highlights the critical role of continuous innovation, strategic alignment, and quality assurance in the development of Medical Information content and KPI reporting. As the industry responds to shifting customer expectations and rapid technological advancement, organizations are required to balance operational efficiency and personalized engagement, as well as innovation with regulatory compliance. Notably, the emerging practice of outsourcing SRD development represents a meaningful shift in operational models. This approach enables internal FTEs to focus more on higher‑value activities, including strategic alignment, cross‑functional collaboration, governance oversight, and quality assurance.

Based on these findings, several recommendations emerge for future actions. Organizations should establish and refine non-traditional content types, processes, and workflows to meet growing customer expectations and support delivery across multiple channels. Companies should expand digital and mobile optimization efforts by prioritizing mobile‑friendly formats and accessibility features to enhance usability for diverse audiences and should invest in digital engagement tracking to better inform content strategy and channel selection. Finally, organizations should strengthen governance around AI and content reuse by creating formal guidelines for AI use and content development, including mandatory human validation to ensure accuracy, consistency, and compliance.

## Supplementary Information

Below is the link to the electronic supplementary material.


Supplementary Material 1



Supplementary Material 2



Supplementary Material 3


## Data Availability

Data is provided within the manuscript or supplementary information files.
