# Supplementary material for: Benchmark Survey on Content Development and Key Performance Indicators: A phactMI Benchmarking Survey Update
Source: Ther Innov Regul Sci. 2026 Jun 15;60(5):1459–67. doi: 10.1007/s43441-026-00999-9 (PMC13526051; doi:10.1007/s43441-026-00999-9)
Supplement: Supplementary file 1 [file 43441_2026_999_MOESM1_ESM.docx]

Benchmark content development and KPI - 2024

Dear phactMI Survey Participant:

Thank you for your time as we conduct our next Benchmark Survey focusing on content Development and Key Performance Indicators (KPIs).

This survey's purpose is to understand current trends in content development and key performance indicators (KPIs) and compare these results to the 2018 survey trends. Your responses to this survey should be based on your Medical Information Department and should be using data from January through December 2023. Please note that there is only one survey sent to each company.

Contact information is for tracking purposes only. All results will be kept confidential. And ad identified aggregate results will be made available. Survey results may be used in future presentations and publications. Thank you.

By clicking next, you agree to continue the survey.

**Company background**

1) Please provide your company name (for tracking purposes only)
2) Please provide an email address for follow-up/clarification on responses (for tracking purposes only)
3) Which describes your company’s Medical Information Department?
   a) US only
   b) US and Global
   c) Global
4) What is the size of your Medical Information Department (excluding call center)?
   a) Internal number of Full-time equivalents (FTEs)
   b) External (contractors or vendor) number of FTEs
5) How is your Medical Information team organized?
   a) By therapeutic area
   b) By franchise
   c) By product
   d) Other (please specify)
6) How many molecular products were supported by the Medical Information  team in 2023?
   a) Products in launch preparation (pre-launch to 6 months post-launch): ___
   b) Current/active products: ___
   c) Mature/established/legacy products: ___
   d) Pipeline products: ___
7) Regarding the content that your Medical Information  team develops, what percentage is for patients/consumers and for health care professionals (HCPs) (percent must equal 100%)
   a) Patient/consumer ___
   b) Healthcare professionals ___
8) Do you create disease-state only response documents (non-product based) for dissemination (either for HCPs or patients)?
   a) Yes
   b) No

If Question 8 answer b, skip to question 16

9) Are the disease-state only response documents for patients or HCPs?
   a) Patients/consumers
   b) Healthcare professionals
   c) Both patients/consumers and healthcare professionals
10) How many disease-state response documents (non-product based) did you create in 2023?
11) Please indicate which formats are utilized for disease state response documents (select all that apply)
    a) Video
    b) Infographics
    c) Interactive (i.e., hyperlinks, other clickable content)
    d) Traditional (static) SRD
    e) Other (please describe)
12) How many times are disease-state response documents used?
    a) Never (created, but never requested)
    b) Rarely (1 – 5 times per month)
    c) Sometimes (6 – 25 times per month)
    d) Frequently (26 – 50 times per month)
    e) Very Frequently (over 50 times per month)
13) Where in the life cycle of the drug was it created?
    a) When the drug is in development (prior to the pre-launch phase)
    b) Pre-launch (just prior to expected FDA approval)
    c) Launch phase (from approval to 6 months post approval)
    d) Post-launch phase (any time after 6 months post approval)
14) What was the impetus for creating them?
15) Who is involved in preparation?

For the reminder of questions on Scientific Response Documents (SRDs), the focus will be on HCP specific documents unless otherwise specified.

16) Which of your staff are involved in the creation, revision and updating of HCP SRD? (Select all that apply)
    a) Contact center representative
    b) Medical information staff (Internal)
    c) External Contractor or Vendor
    d) Other
17) Describe the format of your SRD, (provide percentages, should equal 100%)
    a) Written document with all text (no graphs, charts, figures)
    b) Written document with text, graphs, charts, figures
    c) Short answers (FAQ)
    d) Slide deck
    e) Infographic
    f) Animated PDF
    g) HTML
    h) Video
    i) Plain language summaries
    j) Other

if answer a or b = 0, go to 19

18) What is the average page length of your SRD?
19) Have you adapted your SRDs to mobile content?
    a) Yes
    b) No
20) Describe scientific response document development at your company. Is it centralized or decentralized? (select all that apply)
    a) Creation is centralized - Created by a Global Medical Information then localized at each country/region
    b) Creation is centralized - Created by US Medical Information then localized at each country/region
    c) Creation is centralized - Created by another country/region Medical Information localized as necessary
    d) Creation is decentralized at country/region level (including US)
    e) Other

if a, b, c not selected, go to 23

21) What is the percentage of parent SRDs that are localized/repurposed in your Medical Information Department?
22) What are you localizing? (select all that apply)
    a) Summaries
    b) Format
    c) Label information
    d) Disclaimers
    e) Guidelines/recommendations
    f) Other information (please describe)
23) Do you have an average target time to **create a new SRD (a typical written response document)** in hours (exclusive of external (non-Medical Information) review time)?
    a) Yes (what is it)
    b) No
    c) N/A - we do not create new SRDs
24) Do you have an average target time to **localize a new global SRD (a typical written response document)** in hours (exclusive of external (non-Medical Information) review time)?
    a) Yes (what is it)
    b) No
    c) N/A we do not localize new SRDs
25) Do you have an average target time to **revise/update an SRD (a typical written response document)** in hours (exclusive of external (non-Medical Information) review time)?
    a) Yes (what is it)
    b) No
    c) N/A we do not revise/update SRDs
26) Who routinely reviews your typical written document SRD? Select all that apply
    a) Medical Information Peer review
    b) Medical review
    c) Legal review
    d) Regulatory review
    e) Safety review
    f) Other, please explain

If 26 answer a selected.

27) For Medical Information peer review, please indicate type of review
    a) One peer review (for data check and high-level context)
    b) Two peer reviews, one for data check, one for high level context)

If 26 answer b selected

28) For a Medical review, please indicate who is reviewing the content
    a) Medical Director
    b) Therapeutic Head
    c) Other Medical (please explain)
29) How long (average) does the review time take (specifically the reviewer timeline)?
30) Do you apply an on/off label designation to your SRD? (i.e., disclaimer in SRD, systems designation/meta data field, etc.)
    a) Yes, please provide any comments
    b) No, please provide additional comments
31) Does your review and approval process for SRD differ for on label versus off label?
    a) Yes, please explain
    b) No
32) What features do you include in your SRD? Select all that apply
    a) Summary section
    b) Hyperlinks to sections within the document
    c) Hyperlinks to external sources i.e., package insert, clinical trials, citations, etc.
    d) Graphics beyond a simple table or figures
    e) Audio/video
    f) Table of contents
    g) Reference section
    h) Contact information (in body of SRD, not cover letter)
    i) Disclaimers (in body of SRD, not cover letter)
    j) Other section (please explain)
33) What is the standard expiration date for SRDs?

|  | New product | Mature product | Manufactuing/chemical properties (ingredients) |
| --- | --- | --- | --- |
| a) One year |  |  |  |
| b) Two year |  |  |  |
| c) Three year |  |  |  |
| d) Other |  |  |  |

If other, please explain

34) Do you utilize any content created by other Departments (e.g. Medical Affairs)?
    a) Yes, please explain what kind of content and created by whom
    b) No
35) Are you using any AI and large language models (LLM), such as ChatGPT, for content development?
    a) Yes (please indicate which LLM and how you are using)
    b) No, but are investigating/planning (please explain)
    c) No

[If b or c selected, go to question 37]

36) Is there human validation to the AI generated SRDs?
    a) Yes, please explain
    b) No
37) How are you using LLM for content development (select all that apply)
    a) Writing content
    b) Paraphrasing
    c) Creating images
    d) Creating graphs/charts
    e) Creating infographics
    f) Developing business insights
    g) Other (please explain)

38) Please provide any additional information on your strategy for AI generated SRDs (i.e., how long did it take to implement AI for this purpose from the idea to go live, etc.)
39) Is any Medical Information content integrated into your company’s omnichannel strategy (i.e., use across different channels)?
    a) Yes
    b) No
40) In which of the following channels is Medical Information involved in content development? (select all that apply)
    a) Chatbot
    b) Medical Information Call Center (MICC)
    c) Medical Information website
    d) Live chat
    e) Social media
    f) Interactive Voice Response (IVR)
    g) Tools (I.e. Stability calculators)
    h) Field Team materials (used by field team – slide deck, video, infographic)
    i) Other channels (please describe)
41) Do you use any modular content in your content development?
    a) Yes, structured/component authoring
    b) Yes, tiered modular content
    c) Yes, other please explain
    d) No
42) In developing content, which of the following are incorporated for accessibility features?
    a) Colors used in text or graphics
    b) Fonts
    c) Images (with alternative descriptions provided)
    d) Reader compatibility
    e) None
    f) Other
43) Are you developing a specific type of SRD for specific topics? (Note: digital may include any interactive features including video, hyperlinks, etc.)

Digital SRD	Patients Responses

a) Mechanism of action
b) Clinical trials
c) Safety
d) Dosing and administration
e) Other (please explain)

44) Do you make any changes to content/channels based on customer feedback?
    a) Yes, please explain
    b) No

Custom letter development

45) What percent of your inquiries require a custom response document?
46) Describe your custom response documents
    a) Full letters providing a summary of the literature
    b) Provide reference list only
    c) Other
47) Can a custom letter be reused without review by Medical Information?
    a) Yes
    b) No
48) Who can send out a custom response document once it has been created
    a) Contact Center
    b) Escalation – Tier 2 Medical Information team
    c) Other
49) What is the expiration date for a custom response document?
50) What is the expected targeted time to create a new custom response document in your department in hours?
51) Which of your staff are involved in the creation of custom response letters? Select all that apply
    a) Contact center representative
    b) Medical Information Staff (Internal)
    c) External Contractor or Vendor
    d) Other
52) Who typically reviews custom responses? Select all that apply
    a) No review
    b) Peer review (for data check and high-level context)
    c) Medical review
    d) other, please explain.
53) When is a custom letter converted to a scientific response document?
    a) After receiving a specific number of questions (required #)
    b) Recent data release from a competitor
    c) Other (please explain)

**Key Performance Indicators for Medical Information**

54) Do you differentiate between metrics and KPI?
    a) Yes
    b) No
55) What are your top 5 medical information (including call center) metrics and KPIs reported to senior leadership? (select 5 for each leadership)

|  | Medical Senior Leadership | Senior Leader (outside of Medical Information |
| --- | --- | --- |
| a. Response turnaround time |  |  |
| b. Inquiry volume |  |  |
| c. Service level |  |  |
| d. Abandonment rate |  |  |
| e. Escalation rate |  |  |
| f. Number of documents created/updated |  |  |
| g. Response type (SRD vs. Custom) |  |  |
| h. Number of customer insights reports provided to internal teams |  |  |
| i. Number of promotional pieces reviewed by the team |  |  |
| j. HCP satisfaction with responses |  |  |
| k. Time to fulfil escalated requests |  |  |
| l. Time to fulfil request with SRD |  |  |
| m. Sentiment analysis |  |  |
| n. Trust assessment |  |  |
| o. Impact to patient care |  |  |
| p. Contact types |  |  |
| q. Topics/categories of questions |  |  |
| r. % use of localized SRDs |  |  |
| s. % localization of Global response documents |  |  |
| t. Other (please explain) |  |  |

56) What are your metrics and KPIs specific to customer queries (I.e. call center)?
    a) None
    b) Customer satisfaction surveys
    c) Response/materials relevant
    d) Response turnaround
    e) Inquiry volume
    f) Service level
    g) Abandonment rate
    h) Escalation rate
    i) Response type (SRD versus custom)
    j) Contact types
    k) Topics/categories of questions
    l) Other

[if b not selected go to question 58]

57) Which of the following customer satisfaction metrics do you measure? (select all that apply)
    a) How information was used
    b) Recommend to colleague
    c) Net promoter score
    d) Sentiment analysis
    e) Trust assessment
    f) Impact to patient care
    g) other
58) How do you administer the survey? (select all that apply)
    a) Written response document
    b) Call center
    c) Medical information website
    d) Outsourced market research partner
    e) Advisory Board (live and/ or virtual)
    f) Field medical team
    g) Other
59) To which group(s) do you administer surveys? (select all that apply)
    a) HCPs
    b) Consumers
    c) Other
60) What percentage of **health care professionals** receive information by the following channels?
    a) Verbally on the phone
    b) Digital via search engine
    c) Digital via on site search of medical information website
    d) Chatbot
    e) Voicebot
    f) Email
    g) Regular mail
    h) Fax
    i) Text
    j) Other. Please explain
61) What percentage of **patients** receive information by the following channels?
    a) Verbally on the phone
    b) Digital via search engine
    c) Digital via on site search of medical information website
    d) Chatbot
    e) Voicebot
    f) Email
    g) Regular mail
    h) Fax
    i) Text
    j) Other. Please explain
62) What type of quality assurance monitoring is performed to ensure meeting of metrics and KPIs? Select all that apply
    a) Self-monitoring
    b) Monitoring by Quality Assurance
    c) Monitoring by Pharmacovigilance
    d) Monitoring by Compliance
    e) Other
63) What percentage of Call Center interactions are QA’d per month?
    a) 5%
    b) 10%
    c) Other (please specify)
64) Is there a cap to the number of interactions that are QA’d per month?
    a) Yes (please specify number)
    b) No
65) In your QA procedure do you differentiate between general inquiries and escalated inquiries?
    a) Yes, please explain
    b) No
66) What metrics do you collect based on the source of the inquiry via the digital channels? (select all that apply)
    a) None
    b) Number of times an SRD is accessed via Medical Information website
    c) Number of times an SRD is downloaded via Medical Information website
    d) Number of times an SRD is accessed via phactMI website
    e) Number of times an SRD is accessed via chat
    f) Number of times other assets are accessed
    g) Digital engagement with chat (start with chat vs. Completed with chat)
    h) Digital engagement on website (**time spent** on site)
    i) Google hits
    j) Others (please explain)

[if a, skip to 68]

67) Are you able to differentiate between internal and external uses for these metrics?
    a) Yes
    b) No
68) Does Medical Information capture analytics on the internal utilization of Medical Information assets? (i.e., MSL utilization of SRD, Medical Information documents used in training, M Medical Information documents used in other FAQs developed by others, etc.)
    a) Yes, please explain
    b) No
69) Do any of the above digital metrics impact content development or channel strategy?
    a) Yes, please explain
    b) No
70) Do you measure the patient impact of the information provided?
    a) Yes, please explain how
    b) No
71) Please provide any additional comments or additional context for your responses to the questions on KPI's.
