# Supplementary material for: Benchmark Survey on Content Development and Key Performance Indicators: A phactMI Benchmarking Survey Update
Source: Ther Innov Regul Sci. 2026 Jun 15;60(5):1459–67. doi: 10.1007/s43441-026-00999-9 (PMC13526051; doi:10.1007/s43441-026-00999-9)
Supplement: Supplementary file 2 [file 43441_2026_999_MOESM2_ESM.docx]

Appendix 2. Results by company type

|  | Large | Midsize | Small | P value* |
| --- | --- | --- | --- | --- |
| Content audience:  HCP  Patient | 91%  9% | 87%  13% | 91%  9% |  |
| Disease state content | 18% (2/11) | 37% (3/8) | 36% (5/14) | 0.28 |
| Staff creating, revising, updating SRDs^^^  Contact Center  Med Info  External contractor/vendor  Other departments | 27% (3/11)  73% (8/11)  45% (5/11)  18% (2/11) | 25% (2/8)  100% (8/8)  50% (4/8)  13% (1/8) | 21% (3/14)  100% (14/14)  64% (9/14)  7% (1/14) | 0.77  **0.01**  0.46  0.45 |
| Format of SRD (percentage)  Written doc (all text)  Written doc (text + graphs)  Short answers (FAQ)  Miscellaneous | 21%  56%  7%  15% | 21%  64%  10%  6% | 9%  76%  12%  6% |  |
| SRD length (average pages) | 4.1 | 6.5 | 5.5 | 0.06 |
| SRD adapted to mobile platform | 45% (5/11) | 13% (1/8) | 14% (2/14) | 0.04 |
| Centralized development of SRDs | 91% (10/11) | 88% (7/8) | 79% (11/14) |  |
| Percent parent SRD localized by Med Info | 73% (n=8) | 53% (n=6) | 53% (n=8) |  |
| Sections localized^^^  Summaries  Format  Label information  Disclaimers  Guidelines/recommendations  Other information | 29% (2/7)  14% (1/7)  100% (7/7)  57% (4/7)  71% (5/7)  29% (2/7) | 29% (2/7)  0% (0/7)  100% (7/7)  86% (6/7)  29% (2/7)  14% (1/7) | 45% (5/11)  18% (2/11)  82% (9/11)  64% (7/11)  55% (6/11)  27% (3/11) |  |
| No target time to create new SRD | 60% (6/10) | 72% (5/7) | 86% (12/14) |  |
| No target time to revise/update SRD | 67% (4/6) | 57% (4/7) | 62% (8/13) |  |
| Routine review of SRD  Medical Information Peer review  Medical review  Legal review  Regulatory review  Safety review  Other | 82% (9/11)  55% (6/11)  18% (2/11)  9% (1/11)  9% (1/11)  36% (4/11) | 100% (8/8)  50% (4/8)  13% (1/8)  0  0  13% (1/8) | 71% (10/14) 71% (10/14)  36% (5/14)  21% (3/14)  7% (1/14)  7% (1/14) |  |
| Medical Information peer review  One peer  Two peers | 78% (7/9)  22% (2/9) | 50% (4/8)  50% (4/8) | 60% (6/10)  40% (4/10) |  |
| Medical review  Medical director  Therapeutic head  Other  Medical Information | 43% (3/7)  0  29% (2/7)  29% (2/7) | 43% (3/7)  14% (1/7)  14% (1/7)  29% (2/7) | 58% (7/12)  17% (2/12)  8% (1/12)  17% (2/12) |  |
| Application of on/off label designation | 70% (7/10) | 50% (4/8) | 71% (10/14) |  |
| SRD Expiration date of 1-2 years  New product  Mature product  Manufacturing/Chemical properties | 90% (9/10)  70% (7/10)  70% (7/10) | 100% (7/7)  100% (7/7)  100% (7/7) | 100% (13/13)  77% (10/13)  85% (11/13) |  |
| Features included in SRDs  Summary section  Reference section  Hyperlinks external  Hyperlinks internal  Disclaimers  Graphics beyond simple figure  Table of contents  Audio/video | 91% (10/11)  91% (10/11)  91% (10/11)  73% (8/11)  73% (8/11)  64% (7/11)  45% (5/11)  36% (4/11) | 100% (8/8)  100% (8/8)  75% (6/8  50% (4/8)  50% (4/8)  50% (4/8)  63% (5/8)  0 | 93% (13/14)  93% (13/14)  43% (6/14)  64% (9/14)  57% (8/14)  29% (4/14) 29% (4/14)  0 |  |
| AI and LLM use for content development | 9% (1/11) | 12% (1/8) | 7% (1/14) |  |
| Planned use for AI  Writing content  Paraphrasing  Creating images  Creating Graphs  Creating infographics  Developing business insights  Translation | 100% (5/5)  100% (5/5)  100% (5/5)  80% (4/5)  60% (3/5)  100% (5/5)  0 | 80% (4/5)  100% (5/5)  20% (1/5)  60% (3/5)  40% (2/5)  60% (3 /5)  20% (1/5) | 75% (6/8)  38% (3/8)  0  25% (2/8)  13% (1/8)  13% (1/8)  0 |  |
| Med Info integrated into omnichannel strategy | 82% (9/11) | 57% (4/7) | 43%(6/14) |  |
| Med Info content:  Chatbot  Medical Information Call Center  Medical Information website  Live chat  Social media  Interactive Voice Response (IVR)  Tools (I.e. Stability calculators)  Field Team materials | 27% (3/11)  91% (10 11)  100% (11/11)  27% (3/11)  18% (2/11)  45% (5/11)  45% (5/11)  55% (6 /11) | 57% (4/7)  86% (6/7)  100% (7/7)  29% (2/7)  0  29% (2/7)  43% (3/7)  43% (3/7) | 0  71% (10/14)  79% (11/14)  21% (3/14)  7% (1/14)  0  14% (2 /14) 43% (6/14) |  |
| Use of modular content | 55% (6/11) | 0 | 0 |  |
| Accessibility features  Colors used in graphics or text  Fonts  Images (with descriptions)  Reader compatibility  None | 20% (2/10)  30% (3/10)  40% (4/10)  10% (1/10)  30% (3/10) | 43% (3/7)  14% (1/7)  0  0  57% (4/7) | 38% (5 /13)  23% (3 /13)  31% (4/13)  8% (1/13)  38% (5/13) |  |
| Patient SRDs based on topic  Mechanism of action  Clinical trials  Safety  Dosing and administration | 29% (2/7)  43% (3/7)  43% (3/7)  43% (3/7) | 50% (1/2)  50% (1/2)  50% (1/2)  50% (1/2) | 17% (1/6)  0  17% (1/6)  33% (2/6) |  |
| Digital SRDs based on topic  Mechanism of action  Clinical trials  Safety  Dosing and administration | 100% (7/7)  57% (4/7)  57% (4/7)  71% (5/7) | 50% (1/2)  50% (1/2)  50% (1/2)  50% (1/2) | 67% (4/6)  50% (3/6)  17% (1/6)  33% (2/6) |  |
| Med Info develop Custom Response Documents | 91% (10/11) | 86% (6/7) | 100% (14/14) |  |
| Percent inquiries requiring custom response document | 19% | 25% | 33% |  |
| Description of custom response  Full letter  Reference list  Other | 60% (6/10)  20% (2/10)  20% (2/20) | 50% (3/6)  0  50% (3/6) | 77% (10/13)  15% (2/13)  8% (1/13) |  |
| Dissemination of custom responses^^^  Contact center  Escalation Tier 2 Med info | 40% (4/10)  80% (8/10) | 60% (3 /5)  60% (3/5) | 46% (6/13)  62% (8/13) |  |
| Expiration dates of custom responses  Less than 24 hours  1 – 7 days  1 week – 6 months  1 year  18 months | 25% (2/8)  38% (3/8)  13% (1/8)  25% (2/8)  0 | 0  17% (1/6)  50% (3/6)  33% (2/6)  0 | 55% (6/11)  0  0  27% (3/11)  9% (1/11) |  |
| Target time to create custom document  2-5 hours  `Within 24 hours  Within 48 hours  Within 72 hours  3-5 days | 43% (3/7)  14% (1/7)  29% (2/7)  14% (1/7)  0 | 0  0  50% (1/2)  50% (1/2)  0 | 1-% (1/10)  20% (2/10)  30% (3/10)  20% (2/10)  20% (2/10) |  |
| Staff creating custom responses^^^  Contact center  Medical Information  External vendor | 50% (5 /10)  90% (9/10)  20% (2 /10) | 17% (1/6)  100% (6/6)  50% (3/6) | 7% (1/14)  100% (14/14)  36% (5/14) |  |
| Peer review Custom responses  Peer review  Medical review  Other  No review | 80% (8/10)  20% (2/10)  0  20% (2/10) | 100% (6/6)  50% (3/6)  33% (2/6)  0 | 77% (10/13)  54% (7/13)  23% (3/13)  0 |  |
| Distinction between metrics and KPIs | 64 % (7/11) | 71% (5/7) | 54% (7/13) |  |
| Metrics and KPIs reported to senior leadership^  inquiry volume  Response turnaround time  # documents created/updated  Topics/categories of questions  Escalation rate | 100% (11/11)  73% (8/11)  55% (6/11)  36% (4/11)  55% (6/11) | 100% (6/6)  100% (6/6)  83% (5/6)  67% (4/6)  50% (3/6) | 100% (13/13)  77% (10/13)  69% (9/13)  62% (8/13)  54% (7/13) |  |
| Administration of satisfaction survey^  Written response document  Contact center  Website  Other | 57% ( 4/ 7 )  86% ( 6/ 7 )  57% ( 4/ 7 )  14% ( 1/ 7 ) | 50% ( 3/ 6 )  50% ( 3/ 6 )  33% ( 2/ 6 )  33% ( 2/ 6 ) | 25% ( 2/ 8 )  75% ( 6/8)  13% ( 1/ 8 )  0 |  |
| Metrics customer responses^^^  Inquiry volume  Response turnaround  Customer satisfaction surveys  Service level  Abandonment rates  Escalation rates | 67% (7/11)  55% (6/11)  67% (7/11)  67% (7/11)  67% (7/11)  67% (7/11) | 71% (5/7  71% (5/7)  86% (6/7)  57% (4/7)  57% (4/7)  29% (2/7) | 85% (11/13)  85% (11/13)  62% (8/13)  77% (10/13)  54% (7/13)  54% (7/13) |  |
| Customer satisfaction metrics^  Impact to patients  How information was used  Recommend to colleagues  Other | 67% (4/6)  33% (2/6)  33% (2/6)  50% (3/6) | 80% (4/5)  40% (2/5)  60% (3/5)  40% (2/5) | 25% (2/8)  38% (3 /8)  13% (1/8)  25% (2 /8) |  |
| Survey population  HCP  Patients | 100% (6/6)  67% (4/6) | 100% (6 /6)  50% (3/6) | 100% (8/8)  63% (5/8) |  |
| Channels for HCPs  Phone  digital search engine  Digital Med info website  Chatbot  Email | Average %  29%  2%  11%  1%  55% | Average %  28%  9%  8%  7%  43% | Average %  30%  2%  9%  1%  55% |  |
| Channels for patients  Phone  digital search engine  Digital Med info website  Email | Average %  72%  5%  5%  17% | Average %  60%  0  1%  10% | Average %  63%  6%  1%  21% |  |
| Quality assurance monitoring^  self monitoring  Quality assurance  Pharmacovigilance  Compliance  Other | 82 % (9/11)  36% (4/11)  36% (4/11)  9% (1/11)  0 | 71% (5/7)  57% (4/7)  29% (2/7)  29% (2/7)  0 | 83% (10/12)  25% (3/12)  25% (3 /12)  17% (2/12)  17% (2/12) |  |
| percent interactions contact center monitored monthly  less than 5%  5%  10%  Greater than 10%  Other | 18% (2/11)  18% (2/11)  0  9% (1 /11)  36% (4/11) | 0  14% (1/7)  43% (3/7)  29 % (2/7)  14% (1/7) | 0  38% (5/13)  38% (5 /13)  23% (3/13)  0 |  |
| Cap on inquiries monitored | 18% (2/11) | 0 | 21% (3/14) |  |
| Digital metrics  # SRD accessed website  # SRD downloaded  # SRD via phactMI  # SRD via chat next slide #other # other assets accessed  Digital engagement chat  Digital engagement website  Google hits  Other | 73% (8/11)  55% (6/11)  27% (3/11)  27% (3/11)  27% (3/11)  27% (3/11)  45% (5/11)  9% (1/11)  0 | 100% (5/5)  80% (4/5)  80% (4/5)  60% (3/5)  80% (4/5)  20% (1/5)  40% (2/5)  20% (1/5)  0 | 57% in (8/14)  29% (4/14)  21% (3/14)  0  14% (2/14)  21% (3/14)  0  14% (2/14) |  |
| Digital metrics impact content development or channel strategy | 64% (7/11) | 14% (1/7) | 14% (2 /14) | <0.01 |
| Differentiate between internal and external users | 56% (5/9) | 17% (1/6) | 18% (2/11) | <0.05 |
| Internal users analytics | 45% (5/11) | 0 | 0 |  |

* p value comparing large to the combined midsize and small.

^ select all that apply
