# Supplementary material for: Benchmark Survey on Content Development and Key Performance Indicators: A phactMI Benchmarking Survey Update
Source: Ther Innov Regul Sci. 2026 Jun 15;60(5):1459–67. doi: 10.1007/s43441-026-00999-9 (PMC13526051; doi:10.1007/s43441-026-00999-9)
Supplement: Supplementary file 3 [file 43441_2026_999_MOESM3_ESM.docx]

Appendix 3. Results 2024 vs. 2018

|  | 2024 | 2018 | P value |
| --- | --- | --- | --- |
| Medical Information staff involved in SRD development | 30/33 (91%) | 27/27 (100%) |  |
| Responsible parties for SRD development  Internal FTEs  Both internal and contractors | 17/34 (50%)  17/34 (50%) | 15/27 (56%)  12/27 (44%) |  |
| Staff involved in creation and revision of SRDs  Medical Information  Contact Center  Dedicated content team  Other | 30/33 (91%)  8/33 (24%)  18/33 (55%)  4/33 (12%) | 23/27 (85%)  4/27 (15%)  5/27 (19%)  3/27 (11%) |  |
| Formats for SRDs  Text document  Short answer  HTML  Slide deck  Infographics  Other | 32/33 (97%)  27/33 (82%)  6/33 (18%)  9/33 (27%)  19/33 (58%)  8/33 (24%) | 26/27 (96%)  --  1/27 (4%)  4/27 (15%)  4/27 (15%)  4/27 (15%) |  |
| Average page length of SRDs  2 Pages  3 pages  4 pages  5 pages  6 pages  7 pages  8 - 9 pages  10 or more pages | 1/31 (3%)  8/31 (26%)  4/31 (13%)  7/31 (23%)  3/31 (10%)  3/31 (10%)  5/31 (16%)  1/31 (3%) | 1/24 (4%)  4/24 (17%)  4/24 (17%)  5/24 (21%)  5/24 (21%)  3/24 (13%)  --  2/24 (8%) |  |
| Centralized creation for SRDs | 28/33 (85%) | 22/26 (85%) |  |
| No target time to create a new SRD | 23/32 (72%) | 10/27 (37%) | **<0.01** |
| No target time to revise/update new SRD | 19/32 (59%) | 10/27 (37%) |  |
| Review of SRDs  One peer review  Two peer review  Medical review  Legal review  Regulatory review  Other^ | 17/33 (52%)  10/33 (30%)  20/33 (61%)  8/33 (24%)  4/33 (12%)  8/33 (24%) | 15/27 (56%)  11/27 (41%)  19/27 (70%)  10/27 (37%)  6/27 (22%)  6/27 (22%) |  |
| On/off-label designation on SRDs | 21/32 (65%) | 17/27 (63%) |  |
| No different review process for off-label SRDs | 30/31 (97%) | 25/27 (93%) |  |
| Features included in SRDs  Summary section  Hyperlinks  Graphics beyond simple table/figure  Table of contents  Audio/video | 31/33 (94%)  24/33 (73%)  18/33 (55%)  14/33 (42%)  4/33 (12%) | 25/27 (93%) 18/27 (67%)  8/27 (30%)  4/27 (15%)  4/27 (15%) |  |
| Standard expiration dates for SRDs  One year  Two years  Three years  Variable | 27/33 (82%)  4/33 (12%)  1/33 (3%)  1/33 (3%) | 22/26 (85%)  2/26 (8%)  1/26 (4%)  -- |  |
| Custom letter development – Med Info involved | 29/31 (94%) | 22/26 (85%) |  |
| Staff involved in custom letter development  Medical Information  Contact Center  Dedicated content team  External contractor or vendor | 29/30 (97%)  7/30 (23%)  --  10/30 (33%) | 18/27 (67%)  6/27 (22%)  3/27 (11%)  -- | **<0.01**  0.92  0.06  **<0.01** |
| Review of custom response documents  Peer review  Medical review  Other^#^  No review | 24/29 (83%)  12/29 (41%)  5/29 (17%)  2/29 (7%) | 16/27 (59%)  6/27 (22%)  9/27 (33%)  -- |  |
| Top KPIs reported to senior leadership  Inquiry volume  Response turn around  Service level  Time to fulfill escalated requests | 30/31 (97%)  24/31 (77%)  14/31 (45%)  13/31 (42%) | 13/27 (48%)  16/27 (59%)  4/27 (15%)  3/27 (11%) | **<0.01**  0.14  **<0.05**  **<0.01** |
| Customer satisfaction metrics  How Information was used  Recommend to colleagues  Other yeah | 7/19 (37%)  6/19 (32%)  7/19 (37%) | 7/27 (26%)  11/27 (41%)  8/27 (30%) |  |
| Administration of customer satisfaction survey  written response document  Call center  Medical information website  Outsourced market research partner  Advisory board  Field team  Other | 9 /21 (43%)  15/21 (71%)  7/21 (33%)  --  --  --  3/21 (14%) | 16/27 (59%)  14/27 (52%)  6/27 (22%)  6/27 (22%)  1/27 (4%)  2/27 (7%)  -- |  |
| Groups surveyed for customer satisfaction  HCP only  Consumer only  Both HCP and consumer  Internal customers | 8/20 (40%)  --  12/20 (60%)  -- | 11/27 (41%)  1/27 (4%)  12/27 (44%)  5/27 (19%) |  |

^ other: Depending on the nature of the response document others may include Safety, Editorial, Communications Manager, Quality Assurance, and Contact Center partner.

\# other may include regulatory, clinical development, pharmacovigilance, etc.
